# Disrupting and restoring protection: maternal antibiotic exposure and neonatal IgA transfer via breastfeeding

**DOI:** 10.1038/s41390-025-03992-4

**Published:** 2025-03-11

**Authors:** Olivier Gasser, Gergely Toldi

**Affiliations:** 1 Malaghan Institute of Medical Research, Wellington, New Zealand; 2 Liggins Institute, University of Auckland, Auckland, New Zealand

*A Commentary on “Effect of prenatal antibiotics on breast milk and neonatal IgA and microbiome: a case-control translational study protocol”*

Breast milk (BM) is a highly dynamic, bioactive fluid that provides continuous transfer of maternal microbiota and other compounds, such as human milk oligosaccharides (HMOs), secretory immunoglobulins (IgA, IgG, and IgM), leukocytes, antibacterial factors, microRNA, and stem cells.^1–3^ These compounds indirectly influence microbial colonisation of the infant and contribute to the maturation of the mucosal immune system and systemic immunity.^4^

IgA is the most abundantly produced antibody isotype in humans playing a critical role in mucosal immunity and homeostasis and supports host-microbiome mutualism.^5,6^ The impact of secretory IgA in establishing beneficial microbiota and influencing microbial diversity is significant, with initial exposures via BM and later by endogenous production. Breastfeeding has been linked to reduced infant infection and the risk of chronic disease and allergy in later life, allowing the infant to develop tolerance to antigens.^7,8^ Such activity may be mediated through a transitory supply of the compounds mentioned above, even during the complementary feeding period. These factors, alongside perinatal seeding with maternal microbes, may provide a trajectory for the vertical transmission of protective maternal immunological traits. In mice, longer-lasting immunological adaptations to breastfeeding, mediated through IgA-coated bacteria, have been demonstrated.^9^

As described in the seminal work introducing the IgA-seq methodology in the context of inflammatory bowel disease,^10^ antigen specificity is a crucial attribute of functional IgA responses. Furthermore, the development of the microbiome and associated IgA responses is impacted by BM in an antigen-specific manner, as recently documented in cohorts of mono- and dizygotic twins.^11^ Experimental models additionally suggest that the magnitude of intestinal IgA responses may be maternally transmitted through breastfeeding, across multiple generations.^9^

Appropriate clinical cohorts of mother-baby dyads are crucial in investigating the dynamics of IgA transfer during breastfeeding in humans, and in establishing how the maternal IgA profile might be imprinted in the infant before weaning, improving the specificity of the infant’s own IgA responses, thus contributing to a reduced incidence of infections.^12^

However, antibiotic exposure during pregnancy may disrupt this process by diminishing maternal IgA production and secretion into BM via interfering with the composition of the maternal microbiome. In a recently published mouse study, Pietrasanta et al. demonstrated that antenatal antibiotic exposure increases susceptibility to late onset sepsis, and is associated with lower maternal BM IgA, neonatal fecal IgA, and IgA coating of intestinal bacteria, leading to systemic translocation of intestinal bacteria.^13^ Weaned mice born to antibiotic-treated mothers had reduced intestinal IgA+ plasma cells, fecal secretory IgA, colonic regulatory T cells (Tregs) and Th17 cells, and reduced fecal microbial diversity. However, following antibiotic exposure, treatment of mothers with apyrase, which restores secretory IgA secretion, prompted IgA production in BM and protected pups from sepsis. Additionally, in a cross-fostering experiment, nursing by mothers not exposed to antibiotics rescued the phenotypes of pups born to antibiotic-exposed mothers. These data highlight the impact of prenatal antibiotics on BM IgA and their long-term influence on intestinal mucosal immune phenotype and function of the offspring mediated by breastfeeding.

In the current research protocol,^14^ the authors propose to study the same mechanism in humans. They aim to investigate the following parameters in a prospective case-control cohort of infants exposed or not exposed to prenatal antibiotics: the amount of IgA and the composition of microbiota in BM and neonatal stool, the proportion and profile of IgA-coated bacteria in neonatal stool, and the concentration of an antimicrobial chemoattractant, CCL28 in maternal serum and BM. These parameters will be quantified from samples collected during the first week of life, at one and three months of life, and at 8–12 months of life (or at the time of weaning from breastmilk). A sample size of 41 antibiotic-treated and control women, respectively, was calculated to have sufficient power for this analysis and deemed feasible within the planned one-year study duration.

A limitation of the current protocol is that it will not be adequately powered to analyze the clinical outcomes of the infants enrolled. In the future, larger cohort studies might be able to achieve this goal, focusing on the clinical consequences of prenatal antibiotics on outcomes such as the rates of gastrointestinal and other infections, food allergies or intestinal inflammatory diseases.

Clinically, this study may contribute to advancing selective evaluation of the BM IgA profile and immune phenotype in mothers exposed to prenatal antibiotics. Consequently, the results may help identify correctable factors to improve health outcomes in infants who cannot benefit from maternal BM or who have been antenatally exposed to antibiotics. The findings will clarify the impact of prenatal antibiotics on the immune function of BM in humans, building on the authors’ earlier findings in a mouse study. The trial results will have the potential to influence breastfeeding practices, and may allow infants born following prenatal antibiotic exposure to benefit from interventions improving the immune function of BM.
